# Supplementary figures and images for: Fungal Interactions Strengthen the Diversity-Functioning Relationship of Solid-State Fermentation Systems
Source: mSystems. 2022 Jul 5;7(4):e00401-22. doi: 10.1128/msystems.00401-22 (PMC9426468; doi:10.1128/msystems.00401-22)

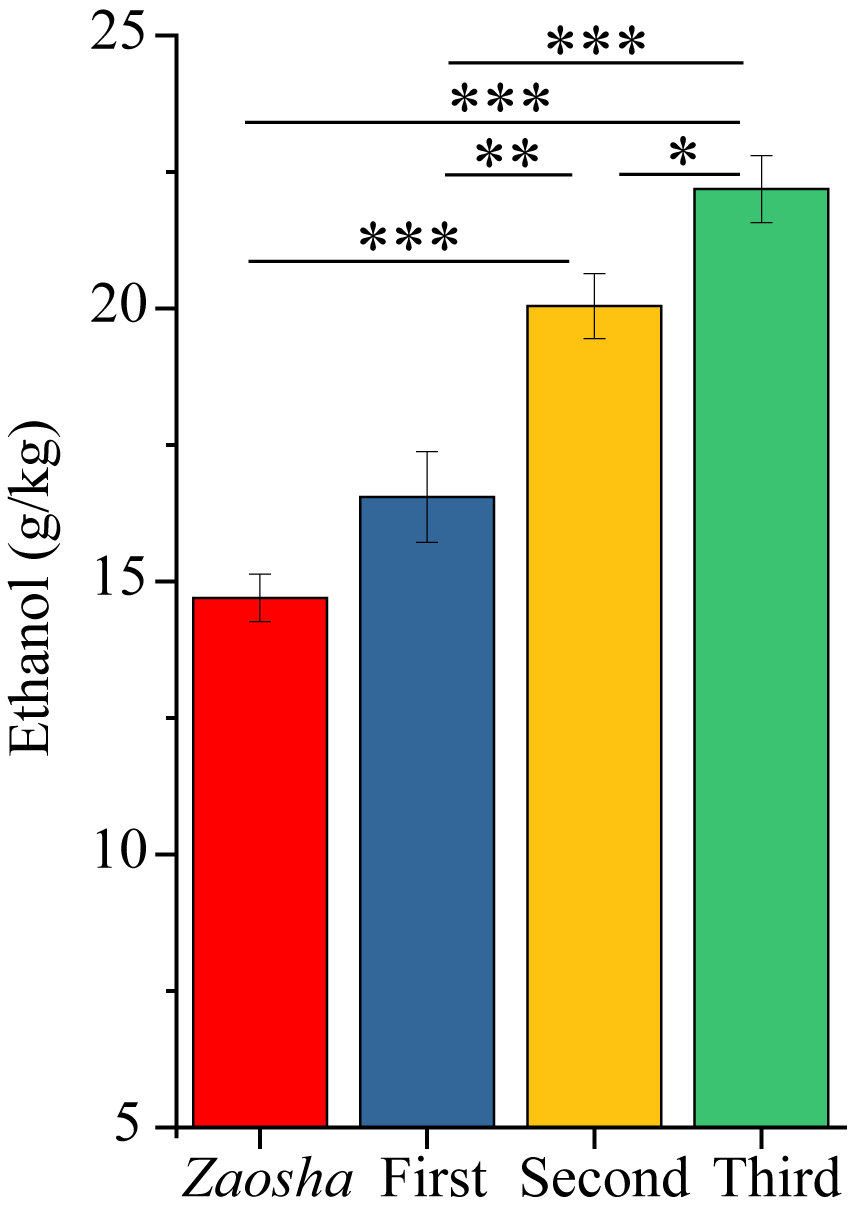

Supplement: FIG S1 [file msystems.00401-22-s0003.tif]

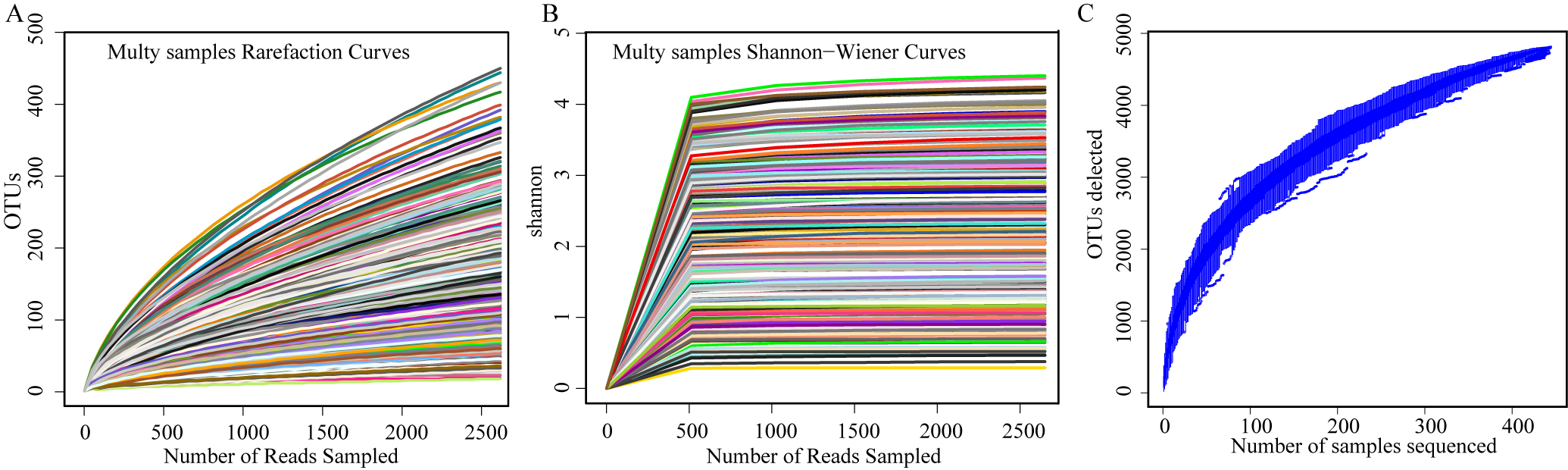

Supplement: FIG S2 [file msystems.00401-22-s0004.tif]

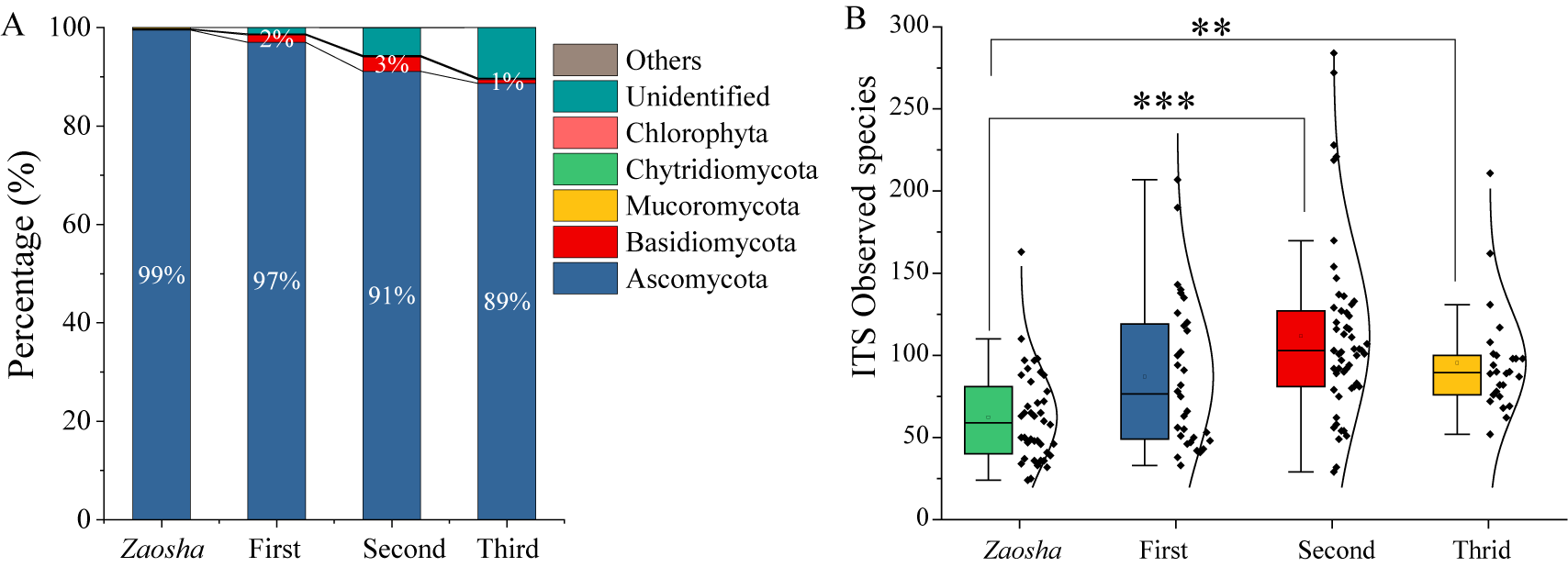

Supplement: FIG S3 [file msystems.00401-22-s0005.tif]

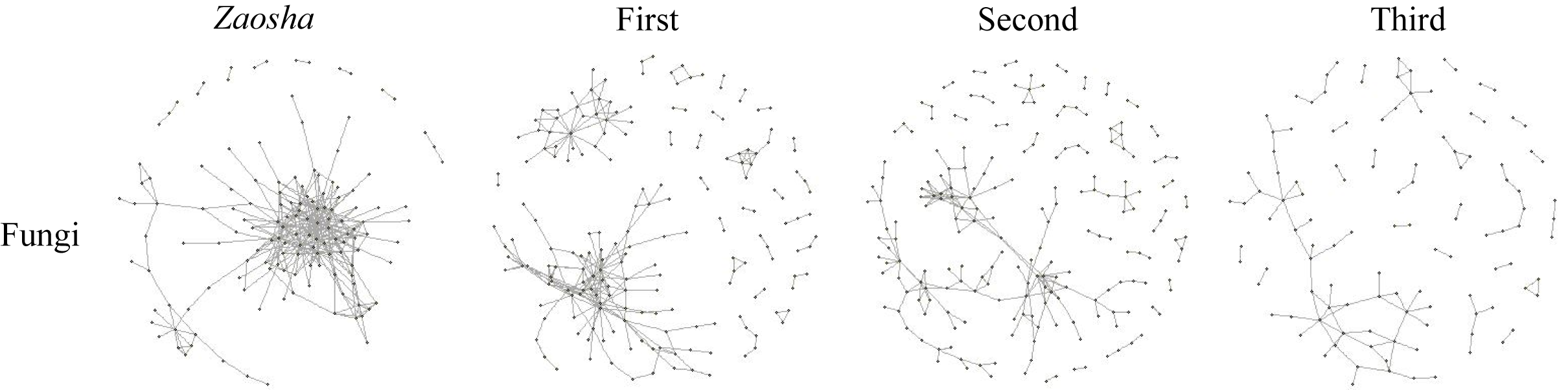

Supplement: FIG S4 [file msystems.00401-22-s0006.tif]

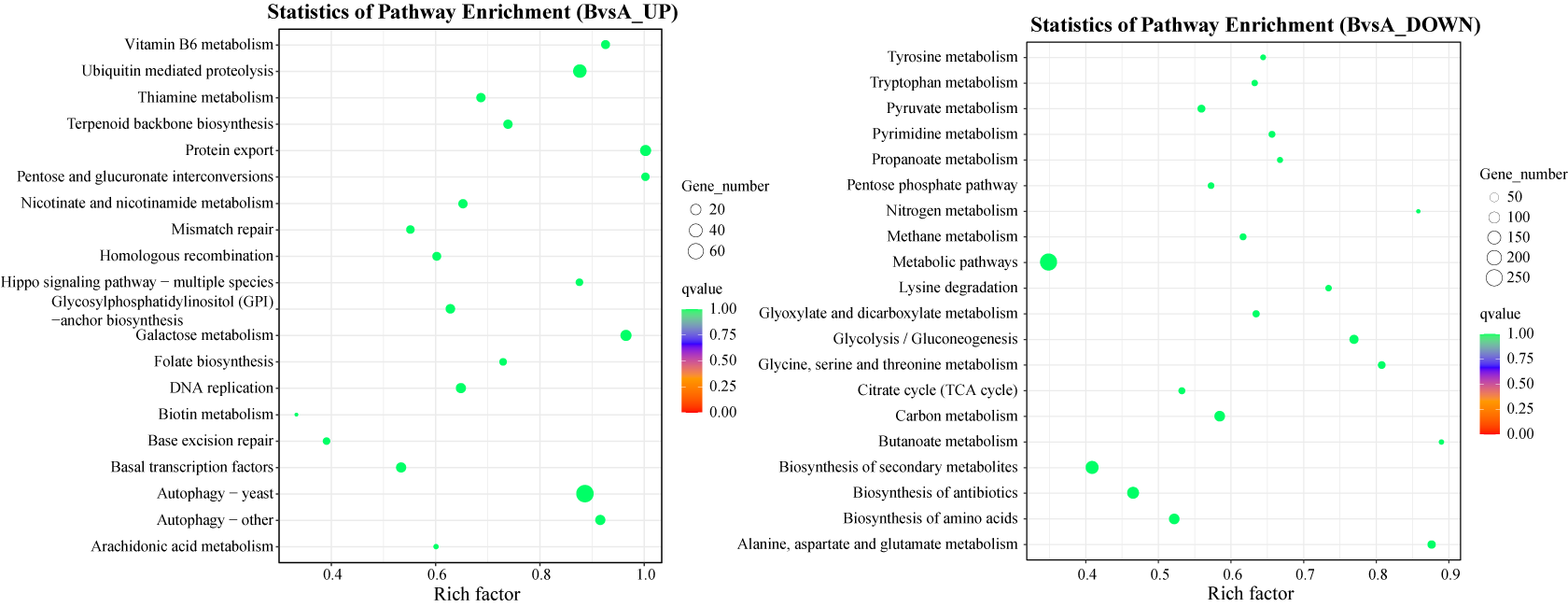

Supplement: FIG S5 [file msystems.00401-22-s0007.tif]

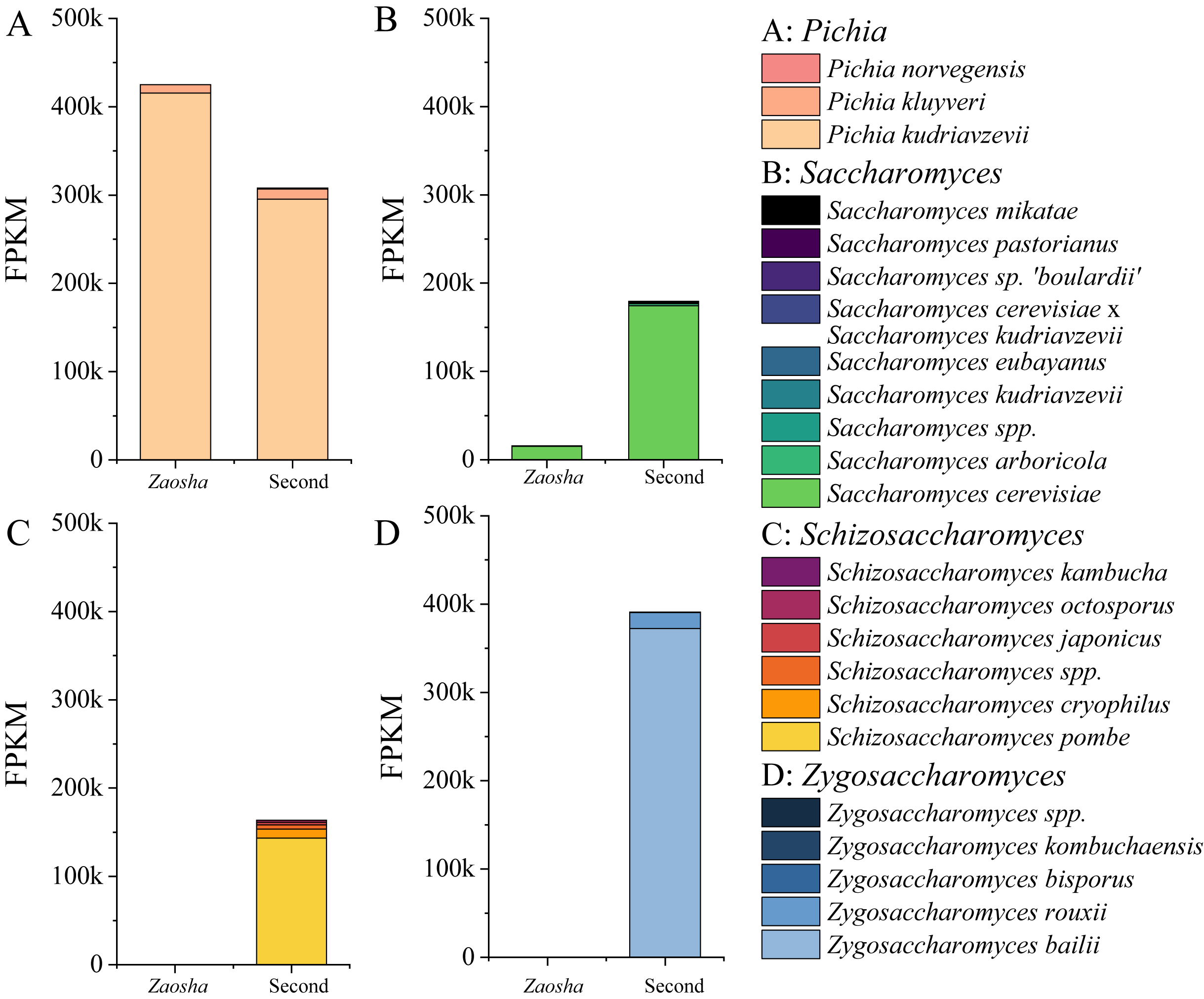

Supplement: FIG S6 [file msystems.00401-22-s0008.tif]
